# MicroRNAs Targeting Caspase-3 and -7 in PANC-1 Cells

**DOI:** 10.3390/ijms19041206

**Published:** 2018-04-16

**Authors:** Jong Kook Park, Andrea I. Doseff, Thomas D. Schmittgen

**Affiliations:** 1 Department of Biomedical Science and Research Institute for Bioscience & Biotechnology, Hallym University, Chuncheon 24252, Korea; 2 Department of Physiology and Department of Pharmacology and Toxicology, Michigan State University, East Lansing, MI 48824, USA; doseffan@msu.edu; 3 Department of Pharmaceutics, College of Pharmacy, University of Florida, Gainesville, FL 32610, USA

**Keywords:** microRNA, caspase-3, caspase-7, TRAIL, pancreatic adenocarcinomas

## Abstract

MicroRNAs (miRNAs), a critical part of the RNA silencing machinery, are known to play important regulatory roles in cancer. However, the consequence of miRNA deregulation in cancer is unknown for many miRNAs. Here, we define that miRNAs, miR-17-5p, miR-132-3p/-212-3p, and miR-337-3p are significantly up-regulated in the pancreatic ductal adenocarcinomas (PDAC) compared to the normal and benign tissues. Furthermore, by using PANC-1 cells, we demonstrate that overexpressed miR-337-3p and miR-17-5p/miR-132-3p/-212-3p can regulate executioner caspases-3 and -7, respectively. In addition, over-expression of miRNAs, especially miR-337-3p, attenuates tumor necrosis factor-related apoptosis-inducing ligand (TRAIL) cytotoxicity in PANC-1 cells. Our findings unveil an important biological function for miRNAs up-regulated in PDAC in coordinately regulating caspases, potentially contributing to the malignant progression of PDAC.

## 1. Introduction

Pancreatic ductal adenocarcinoma (PDAC) is an aggressive and incurable malignancy that is anticipated to be the second leading cause of cancer-related deaths by the year 2030 [1]. Many patients with PDAC possess lymph node or distant metastases upon diagnosis. Several signaling pathways involved in growth and proliferation are activated in PDAC. Mutations in tumor suppressor genes are also detected in PDAC [2]. Despite much progress on pancreatic cancer research over the past several decades, effective treatment regimens are lacking. Chemotherapy and radiation therapy remain mostly ineffective [3]. Surgery remains the only attempt at a curative resection, but only 15–20% of PDAC patients are eligible for surgery at the time of presentation [4]. A better understanding of the molecular pathogenesis of PDAC is required to develop novel treatment strategies to combat this disease.

Apoptosis is generally regulated by caspases, a family of cysteine proteases. Activation of initiator pro-caspases, triggered by various stimuli, cleaves executioner pro-caspases by which effective caspases catalyze the cleavage of specific substrates, resulting in chromatin condensation and nuclear fragmentation with the formation of apoptotic bodies [5]. FADD-like ICE inhibitory proteins (FLIP) and the inhibitor of apoptosis (IAP) family, including cIAP, XIAP, and survivin, inhibit the activation of pro-caspases leading to cancer resistance toward tumor necrosis factor-related apoptosis-inducing ligand (TRAIL) induced cell death [6]. Accumulating evidence has shown the importance of executioner caspases. Caspase-3- and/or caspase-7-deficient mouse embryonic fibroblasts showed resistance to apoptosis mediated by FasL and TNF-α [7]. Resistance to apoptosis was reported in MCF-7 human breast carcinoma cells that lack expression of caspase-3 [8]. In addition, TRAIL induced apoptosis is attenuated by caspase inhibitors [9].

microRNAs (miRNAs) are expressed in a cell- and tissue-specific manner and are differentially expressed in cancers [10,11,12,13]. miRNAs may function as oncogenes or tumor suppressors depending upon their expression level, target genes, and the type of cancer. Our laboratory [14] and others [15,16] have demonstrated that miRNAs are differentially expressed in clinical specimens of PDAC. These studies showed that a large number of miRNAs are differentially expressed in PDAC, with a predominately increased expression in the tumor compared to the normal pancreas, chronic pancreatitis, and adjacent benign tissue. In our present study, we investigated whether executioner caspases, caspase-3, and caspase-7, are directly targeted by miR-337-3p, miR-17-5p, and miRs-132-3p/-212-3p in PANC-1 cells. Overexpression of miRNAs targeting executioner caspases attenuates TRAIL cytotoxicity in pancreatic tumor cells.

## 2. Results

### 2.1. miRNA Expression in PDAC Specimens

**Figure 1:**
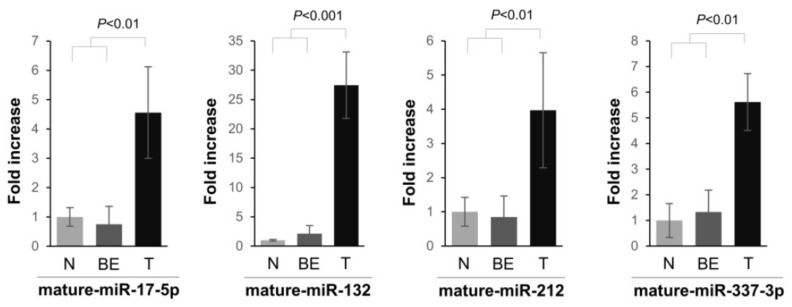
Expression levels of mature miRNAs, miR-17-5p, miR-132-3p, miR-212-3p, and miR-337-3p in normal (N), benign (BE), and malignant (T) human pancreatic tissues. The expression of miRNAs was quantified using the TaqMan MicroRNA Assay. miRNA expression was related to the 18S ribosomal RNA internal control, and data were calculated using the comparative C_T_ method. The relative fold increase of miRNAs with respect to normal tissues are presented.

We previously employed a high throughput miRNA precursor profiling analysis to identify PDAC-related miRNAs. It was demonstrated that a large number of precursor and mature miRNAs were aberrantly expressed in PDAC [14]. The real-time qPCR assay was performed here to validate selected mature miRNA levels. The expression of four miRNAs was validated in 21 pancreas specimens (4 normal pancreas specimens, 6 adjacent benign tissues, and 11 PDACs). Mature miRNAs, miR-17-5p, miR-132-3p, miR-212-3p, and miR-337-3p, were significantly upregulated in PDAC compared to the normal and benign tissues (Figure 1). The expression of mature miR-17-5p was significantly up-regulated (4.4-fold) in PDAC versus normal and benign tissues. miR-132-3p and miR-212-3p were significantly up-regulated in all 11 PDAC, versus normal and benign, tissues, and the average fold increase of each miRNA was 26.1 and 3.9, respectively (Figure 1). In addition, there was an approximately five-fold increase in mature miR-337-3p expression in the PDAC compared to normal and benign tissue (Figure 1).

### 2.2. Identifying Targets of Differentially Expressed miRNAs

**Figure 2:**
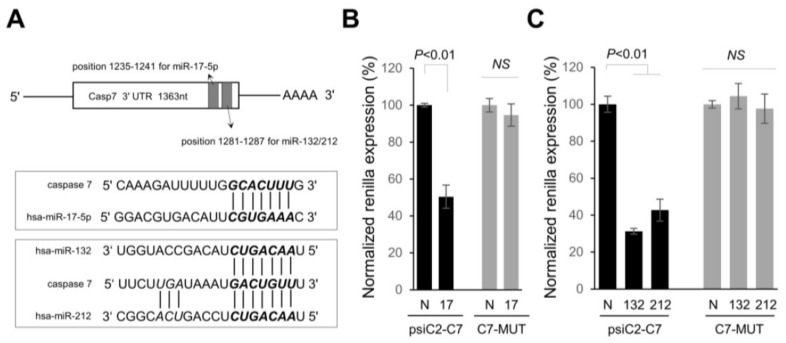
Caspase-7 (casp-7) is directly targeted by miR-17-5p and miR-132-3p/-212-3p in pancreatic tumor cells. (**A**) Schematic representation showing the location of putative miR-17-5p and -132-3p/-212-3p binding sites harbored in the 3′UTR of casp-7 mRNA. (**B**) Luciferase reporter plasmids carrying a wild-type (psiC2-C7) or mutant binding sequences in 3′UTR of casp-7 (C7-MUT) were co-transfected in PANC-1 cells with the negative control of pre-miR precursor (N) or miR-17-5p precursor (17) at 50 nM concentration. At 24 h after transfection, luciferase activity was measured using dual-luciferase assays. Normalized Renilla luciferase activity in cells transfected with N was set at 100%. The data are the mean ± s.d. of at least 3 independent experiments. (**C**) Wild type (psiC2-C7) or mutant plasmids were co-transfected in PANC-1 cells with the negative control of pre-miR precursor (N), miR-132-3p precursor (132) or miR-212-3p precursor (212) at 50 nM concentration. At 24 h after transfection, luciferase activity was measured using dual-luciferase assays. Normalized Renilla luciferase activity in cells transfected with control oligo (N) was set at 100%. The data are the mean ± s.d. of at least 3 independent experiments. NS, not significant.

**Figure 3:**
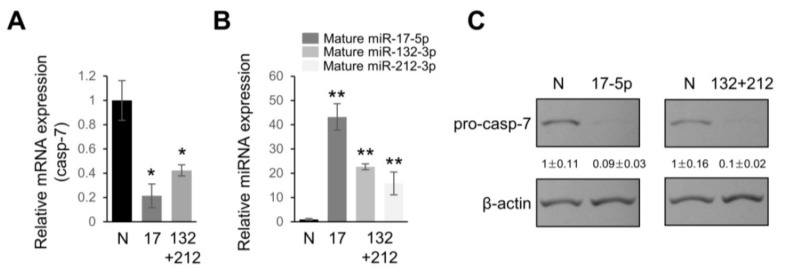
Caspase-7 (casp-7) is downregulated in miR-17-5p and miR-132-3p/-212-3p overexpressing cells. (**A**) Real time qPCR analyses of casp-7 mRNA levels in PANC-1 cells transfected with pre-miR-negative control (N), pre-miR-17-5p, or a mixture of pre-miR-132-3p and -212-3p (25 nM + 25 nM) at 50 nM for 48 h. The data are the mean ± s.d. for three independent experiments. * *p* < 0.01. (**B**) Treatment conditions were the same as in (**A**) and the expression levels of either miR-17-5p, miR-132-3p, or miR-212-3p in PANC-1 cells were determined by real time qPCR. ** *p* < 0.001. (**C**) PANC-1 cells were transfected with pre-miR-negative control (N), pre-miR-17-5p, or a mixture of pre-miR-132-3p and -212-3p (25 nM + 25 nM) at 50 nM for 48 h, and then protein lysates were isolated and subjected to Western blot analysis of pro-casp-7. β-actin served as a loading control and relative expressions are indicated. Western blotting and qPCR were performed on separately treated samples of cells.

To uncover the contribution of overexpressed miRNAs in PDAC to the malignant phenotype, we used in silico and cell-based luciferase assays to find a common target. The TargetScan algorithm was used to look for potential targets. Interestingly, it was found that miR-17-5p and miRs-132-3p/212-3p have miRNA: mRNA predicted interactions for the 3′ untranslated region (UTR) of casp-7 mRNA (Figure 2A). Caspase-7 contributes to the cleavage of substrates that takes place during programmed cell death under certain environments. For example, activation of caspase-7 involves poly(ADP-ribose) polymerase (PARP) cleavage in MCF-7 cells lacking caspase-3 [17,18]. We demonstrated that caspase-7 was a bona fide target of miRs-17-5p, and miR-132-3p/-212-3p. Luciferase activity in PANC-1 cells, transfected with vectors harboring 3′UTR of caspase-7 (psiC2-C7), showed a significant reduction with pre-miR-17-5p, pre-miR-132-3p, and -212-3p when compared with the negative control (Figure 2A). In contrast, no significant change was observed with a vector mutated in the seed-match sequence lacking the 5′ miRNA-end complementarity sites (Figure 2). We also used a miRNA mimic approach to overexpress these miRNAs in PANC-1 cells and noted a significant decrease in caspase-7 mRNA and protein levels compared to the negative control, demonstrating that miR-17-5p, -132-3p, and -212-3p downregulate caspase-7 proteins partly via mRNA degradation (Figure 3). It should be noted that we did not see evidence of cleavage (activation) of the caspase-7 precursor by over-expressing miR-17-5p, -132-3p, and -212-3p in PANC-1 cells.

### 2.3. miR-337-3p Directly Targets Caspase-3

**Figure 4:**
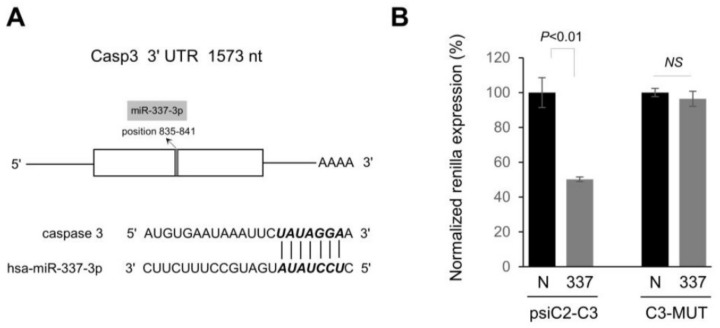
Caspase-3 (casp-3) is directly targeted by miR-337-3p. (**A**) Schematic diagram of human casp-3 mRNA and the predicted target site of miR-337-3p in the 3′UTR is shown. (**B**) Luciferase reporter plasmids carrying a wild-type (psiC2-C3) or mutant binding sequences in 3′UTR of casp-3 (C3-MUT) was transiently co-transfected in PANC-1 cells with pre-miR-negative control (N), or miR-337-3p precursor (337) at 50 nM concentration. At 24 h after transfection, luciferase activity was measured using dual-luciferase assays. Normalized Renilla luciferase activity in cells transfected with control oligo (N) was set at 100%. The data are the mean ± s.d. of at least 3 independent experiments. NS, Not significant.

**Figure 5:**
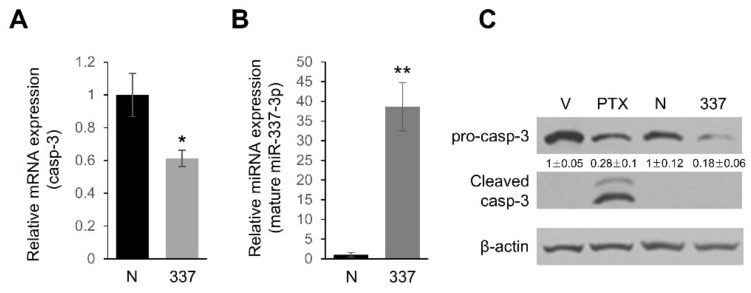
Caspase-3 (casp-3) is downregulated in miR-337-3p overexpressing cells. (**A**) The level of casp-3 mRNA was determined by real time qPCR and normalized with the 18S RNA level in PANC-1 cells after transfection of pre-miR-negative control (N) or pre-miR-337-3p (337). * *p* < 0.01. (**B**) Treatment conditions were the same as in (**A**) and the expression level of mature miR-337-3p in PANC-1 cells was determined by real time qPCR. ** *p* < 0.001. (**C**) Either pre-miR-nega control (N) or pre-miR-337-3p (337) was transfected into PANC-1 cells at 50 nM for 48 h, and then protein lysates were subjected to Western blot analysis of pro-casp-3 and cleaved casp-3. Paclitaxel (PTX) treated (1000 nM for 48 h) PANC-1 cells were used as a positive control of cleaved casp-3 expression. β-actin served as a loading control and relative expression are indicated. V indicates vehicle (DMSO) treated cells. Western blotting and qPCR were performed on separately treated samples of cells.

With respect to programmed cell death, caspase-3 is one of the specific effector caspases [17]. Therefore, we concentrated our efforts on whether caspase-3 is also targeted by a miRNA over-expressed in PDAC. Among several candidate miRNAs, we found that miR-337-3p has miRNA: mRNA-predicted interactions for the 3′UTR of caspase-3 mRNA (Figure 4A). To experimentally validate if the 3′UTR of caspase-3 mRNA indeed contains matching sites for the interaction with miR-337-3p, we carried out a luciferase assay. Transfection of pre-miR-337-3p into PANC-1 cells along with a reporter construct, harboring the 3′UTR of caspase-3, strongly reduced luciferase expression, whereas no significant effect was observed with a construct containing the 3′UTR of caspase-3 mRNA where three core binding sites for miR-337-3p were mutated (Figure 4B). Real time qPCR analysis showed that the caspase-3 mRNA level was decreased by approximately 40% with an overexpression of miR-337-3p (Figure 5A). A significant reduction in pro-caspase-3 protein was noted in PANC-1 cells transfected with pre-miR-337-3p compared with miRNA negative controls without any detection of activated (cleaved) caspase-3, suggesting caspase-3 expression can be downregulated by both mRNA degradation and translational repression (Figure 5B).

### 2.4. Overexpression of miRNAs Targeting Caspases-3/7 Diminishes TRAIL Effects on PANC-1 Cells

**Figure 6:**
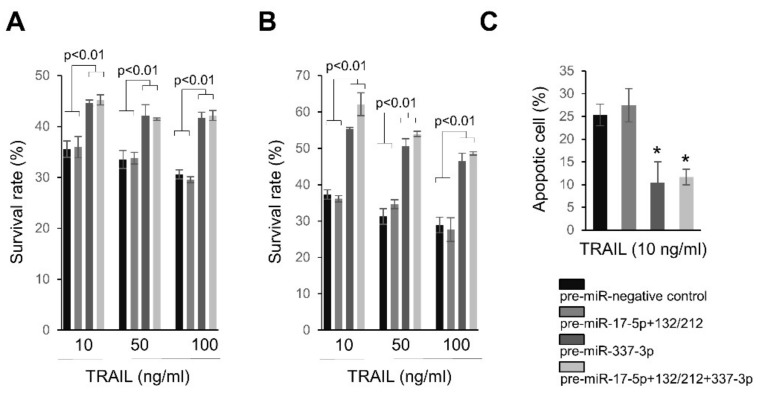
Ectopic expression of miRNAs targeting caspases-3 and caspase-7 renders PANC-1 cells resistant to TRAIL. (**A**) PANC-1 cells were transfected either pre-miR-nega (100 nM), pre-miR-17-5p (50 nM) + a mixture of pre-miR-132-3p and -212-3p (50 nM), pre-miR-337-3p (100 nM), or pre-miR-17-5p (25 nM) + a mixture of pre-miR-132-3p and -212-3p (25 nM) + pre-miR-337-3p (50 nM) in 96 well plates. At 72 h after transfection, TRAIL was treated for 24 h followed and survival rates were measured using WST1 assay. (**B**) Treatment conditions were the same as in (**A**) except that final concentrations of oligos were 150 nM (pre-miR-negative control (150 nM), pre-miR-17-5p (75 nM) + a mixture of pre-miR-132-3p and -212-3p (75 nM), pre-miR-337-3p (150 nM), or pre-miR-17-5p + a mixture of pre-miR-132-3p and -212-3p (75 nM) + pre-miR-337-3p (75 nM)) in 96 well plates. (**C**) Under same transfection conditions in (**B**), apoptotic cells were evaluated by Annexin-FITC assay 12 h after treatment 10 ng/mL TRAIL in PANC-1 cells. * *p* < 0.001.

Based on our findings, we further examined the effects of miRNA targeting caspase-3 and -7 on the antiproliferative effects of TRAIL. PANC-1 cells over-expressing miR-337-3p became resistant to various concentrations of TRAIL, as compared to negative controls or pre-miR-17-5p+miR-132-3p/-212-3p transfected cells (Figure 6A,B). However, under our experimental conditions, there was no further contribution to TRAIL resistance when PANC-1 cells were co-transfected with miR-337-3p and miR-17-5p+miR-132-3p/-212-3p (Figure 6A,B). Following the treatment of TRAIL, the annexin-FITC assay showed decreased apoptotic events in PANC-1 cells over-expressing miR-337-3p, regardless of the over-expression of miR-17-5p+ miR-132-3p/-212-3p (Figure 6C).

### 2.5. Gene Expression Correlation in TCGA Data Set

We collected miRNA and mRNA gene expression data on 139 PDAC tumor samples from the Cancer Genome Atlas (TCGA) database to analyze potential correlations between the differentially expressed miRNAs and their target genes. An inverse correlation was observed between caspase 7 and both miR-132-3p/miR-212-3p confirming our in vitro data (Supplementary Figure S1). Although our cancer cell line data demonstrated that miR-17-5p targets caspase 7 and miR-337-3p directly targets caspase 3 mRNA, there were no inverse correlations between miR-337-3p and caspase-3 or miR-17-5p and caspase 7 in the TCGA data (Supplementary Figure S1).

## 3. Discussion

We report here several miRNAs that regulate caspase-3 and caspase-7 in PANC-1 cells. Using luciferase reporter assays, Western blotting and real-time qPCR, we demonstrated that both miR-17-5p and miR-132-3p/-212-3p directly target caspase-7. In a similar manner we have shown that caspase-3 mRNA is regulated by miR-337-3p. miR-132-3p and miR-212-3p are located on chromosome 17p13.3, approximately 260 bp from each other. Both miR-132-3p and miR-212-3p share the identical 5′ seed sequence. miR-132-3p expression is increased in many types of cancer including lung [19] and colorectal cancer [20]. Increased miR-212-3p expression was also reported in colorectal carcinoma [21]. Several cancer-related targets of miR-132-3p/-212-3p have been validated including MECP2, heparin binding epidermal growth factor EGF, and p120RasGAP [22,23,24]. Our previous study also demonstrated that an important tumor suppressor, Rb1, is a target of miR-132-3p/-212-3p in PDAC. Downregulation of Rb expression by miR-132-3p/-212-3p led to enhanced cell proliferation and cell cycle progression in PANC-1 cells [25]. Recently, it was also demonstrated that inhibition of miR-132-3p/-212-3p causes apoptosis in neuronal apoptosis by targeting FOXO3a [26]. In addition, several reports have demonstrated that several genes such as HBP1 [27], E2F1 [28], and RBL2 [29] are direct targets of mir-17-5p, one of the miR-17-92 polycistrons, a cluster of seven miRNAs derived from the c-myc regulated c13orf25 locus at chromosome 13q31.3.

Apoptosis can be induced by caspase-dependent and/or -independent pathways. Since it has been evident that miRNAs directly regulate gene expression, several reports have demonstrated miRNA-mediated cell death or resistance to therapeutics through targeting oncogenes or tumor suppressor genes. Bcl-2 is known to be regulated by miR-15b and miR-16 [30]. miR-145 targets core binding factor beta subunit (CBFB), protein phosphatase 3, catalytic subunit, alpha isozyme (PPP3CA), and clathrin interactor 1 (CLINT1), resulting in caspase-dependent and -independent cell death in urothelial cancer cells [31]. To our knowledge, only a few reports have revealed the miRNA-mediated direct regulation of caspase expression. Overexpression of let-7a resulted in the resistance of A431 and HepG2 cells to apoptosis induced by interferon-gamma, doxorubicin, and paclitaxel through caspase-3 downregulation [32]. It was also demonstrated that miR-133 is preferentially expressed in cardiac and skeletal muscle and represses caspase-9 expression at both the protein and mRNA levels [33]. In addition, miR-24a also targets caspase-9 [34].

The contribution of each executioner caspase to apoptosis still remains to be uncovered. Actually, caspase-6 and -7 are regarded as executioner caspases, but their role during the phase of apoptosis is ambiguous [35]. For example, caspase-7 has been reported to cleave poly ADP ribose polymerase (PARP), but cleavage of this substrate can also take place in the absence of caspase-7 [18]. Although caspase-7 is known to be redundant to caspase-3, caspase-7 is also considered to regulate cell cycles, cell proliferation, and differentiation [36,37,38]. Therefore, our finding of no additional protective effects of miR-17-5p and miR-132-3p/-212-3p in PANC-1 cells on TRAIL cytotoxicity is indicative that caspase-7 may have a less important role in apoptosis induction in PANC-1 by TRAIL treatment. It is also possible that the targeting of both miR-17-5p and miR-132-3p/-212-3p can contribute to overall cell survival after treatment of TRAIL. Caspase-3 is thought to be the dominant executioner caspase. Activation of caspase-3 requires proteolytic processing and is necessary for cleavage of substrates, DNA fragmentation, and nuclear collapse [35]. We demonstrated that miR-337-3p directly regulates caspase-3 expression and affects TRAIL cytotoxicity in PANC-1 cells. It was reported that miR-337 inhibits proliferation and invasion of pancreatic tumor cells via targeting HOXB7 [39]. Since caspase-3 is also considered to induce ERK activation resulting in the enhanced metastasis of tumor cells in a protease-independent mechanism [40], our finding of the caspase-3 regulation by miR-337-3p suggests the possibility that targeting caspase-3 by miR-337-3p may contribute to cell invasion in the absence of external stimuli.

We report here that multiple miRNAs, miR-17-5p, miR-132-3p/-212-3p, and miR-337-3p directly regulate the expression of caspase-3 and -7. While the direct regulation of the mRNAs by these miRNAs was demonstrated in vitro, inverse correlations between the miRNA and mRNA in patient’s specimens were observed only for miR-132-3p/-212-3p and caspase 7. Several explanations are possible for the discrepancy between the observed in vitro data and those obtained from RNA sequencing experiments, as suggested by Giles et al. [41]. These include greater translational repression in vivo rather than transcriptional repression, and the fact that the mRNA may be targeted by multiple miRNAs and that another layer of regulation exists that is not accounted for by simple correlations. The consequence of the interactions between the miRNAs and caspase mRNA reported here includes the modulation of TRAIL cytotoxicity in pancreatic tumor cells. Since caspases have multifaceted roles in cells, our findings suggest a novel mechanism for the understanding of PDAC progression. Our observation may also serve to clarify the role of these miRNAs in other cancer types and related diseases.

## 4. Materials and Methods

### 4.1. Tissue Procurement

The tissue samples analyzed in this study were derived from patients undergoing a surgical procedure to remove a portion of the pancreas at the University of Oklahoma Health Sciences Center. The collection of samples conformed to the policies and practices of the facility’s Institutional Review Board. Pathology faculty performed a gross analysis of the specimen and selected cancerous-appearing pancreatic tissue and normal-appearing pancreatic tissue for research. Each sample was placed in a cryovial and flash-frozen in liquid nitrogen, and stored at −150 °C until analysis. Subsequent pathologic analysis by the institutes providing the surgical specimens confirmed the histopathology of the samples taken for research. Clinical data on these specimens have been previously reported by us [14,42], and are summarized in Table S1.

### 4.2. Cell Lines and Culture

The human pancreatic cancer cell line, PANC-1, was purchased from the American Type Tissue Collection (Manassas, VA, USA). PANC-1 cells were grown in Dulbecco’s Modified Eagle Media (Invitrogen, Carlsbad, CA, USA) with l-glutamine (Invitrogen) and 10% heat-inactivated fetal bovine serum (FBS, HyClone, Pittsburg, PA, USA, Fisher Scientific). The cells were incubated at 37 °C under a humidified atmosphere with 5% carbon dioxide.

### 4.3. qPCR for miRNA and mRNA Expression

One hundred nanograms of total RNA was primed using gene-specific looped primers with a 20 μL reaction volume, and the cDNA was quantified with TaqMan miRNA Assays (Applied Biosystems, Foster City, CA, USA). For mRNA expression analysis, cDNA was synthesized from 1 µg of total RNA using random primers with a final volume of 25 μL. Gene expression analysis was performed by qPCR using the SYBR^®^ Green PCR Master Mix (Applied Biosystems) according to the manufacturer’s instructions. For qPCR of both miRNA and mRNA, 18S rRNA was used as the reference gene and data were analyzed using the comparative C_T_ method. Both miRNA and mRNA were considered expressed in a particular sample if the mean C_T_ ≤ 35 and they were considered differentially expressed if the fold change between the comparative groups was greater than 1.5-fold and *p* < 0.05 (Student’s *t*-test). Primer sequences were as follows: Caspase 3, sense 5′-TGGATTATCCTGAGATGGGTTT-3′ and antisense 5′-TTGCTGCATCGACATCTGTA-3′; Caspase 7, sense 5′-ACTGCTCTTGTGCCAAGATG-3′ and antisense 5′-CATGGCTTAAGAGGATGCAG-3′; 18S, sense 5′-GTAACCCGTTGAACCCCATT-3′ and antisense 5′-CCATCCAATCGGTAGTAGCG-3′.

### 4.4. Luciferase Reporter Constructs and Assay

In order to identify the miRNA regulating caspase-3 expression, we undertook a bioinformatics approach (Targetscan, miRanda, RNAHybrid) for putative mRNA targets. Either a 1420 bp fragment encompassing a portion of the human caspase-3 3′UTR (accession number NM_004346; the entire length is 1573 bp) or a 1357 bp fragment encompassing a portion of the human caspase 7′ UTR (accession number NM_001227; the entire length is 1363 bp) was PCR amplified using an AmpliTaq^®^ DNA Polymerase (Applied Biosystems). The fragments were subsequently gel purified, and ligated into the psiCHECK-2 vector (Promega, Madison, WI, USA) between the XhoI and NotI sites, located downstream of the Renilla luciferase reporter gene. The mutant reporter constructs of caspase-3 (C3-MUT) and caspase-7 (C7-MUT-1 for miR-132/-212 and C7-MUT-2 for miR-17-5p) were generated by utilizing the wily type psiCHECK-2 vector as a template. Mutating the first three nucleotides of the seed-match sequence was accomplished using the QuikChange site-directed mutagenesis kit (Stratagene, La Jolla, CA, USA). Reporter vectors were assayed for luciferase expression using the Dual Luciferase Report Assay System (Promega) following the manufacturer’s instructions. Primer sequences were as follows: Caspase 3 3′UTR, sense 5′-ATGCCTCGAGAGAAATGGTTGGTTGGTGGTT-3′, and antisense 5′-ATTAGCGGCCGCAATGCGAGCTCATTTTTTGA-3′; Caspase 7 3′UTR, sense 5′-ATGCCTCGAGCAGGGGTACATTCTAGCTGAG-3′, and antisense 5′-ATTAGCGGCCGCAGGAATTAAGCAACCACATTT-3′; C3-MUT, sense 5′-AGGTAATGTGAATAAATTCTATAACAACATATGAAAATAC-3′, and antisense 5′-GTATTTTCATATGTTGTTATAGAATTTATTCACATTACCT-3′; C7-MUT-1, sense 5′-GAATTCTTGATAAATGACTACATTTTTCTGCCTAATAGTA-3′, and antisense 5′-TACTATTAGGCAGAAAAATGTAGTCATTTATCAAGAATTC-3′; C7-MUT-2, sense 5′-TGGCAAAGATTTTTGGCACCACGTTTTCAAGATGGTGT-3′, and antisense 5′-ACACCATCTTGAAAACGTGGTGCCAAAAATCTTTGCCA-3′.

### 4.5. microRNA Transfections

Pre-miR microRNA precursors and control oligomers were purchased from Ambion. To transfect cells with microRNA precursor or control oligo, cells were plated 1 day before transfection, and were transfected using Lipofectamine 2000 and Opti-MEM medium (Invitrogen) following the manufacturer’s protocol.

### 4.6. Protein Extraction and Immunoblotting

Protein was harvested using a RIPA buffer (Pierce, Rockford, IL, USA) and 1× protease and phosphatase inhibitors using standard techniques, and protein concentration was measured using the bicinchoninic acid (BCA) Protein Assay Kit (Fisher Scientific, Pittsburg, PA, USA). Twenty to thirty micrograms of total protein extract were separated on a 10% sodium dodecyl sulfate polyacrylamide gel electrophoresis (SDS-PAGE). Blotting was performed for procaspase-3 (31A893) (Abcam, Cambridge, UK), cleaved caspase-3 (Cell Signaling, #9662), and caspase-7 (Cell Signaling, #9492). β-actin (Abcam, Cambridge, UK) was used as a loading control. The secondary horseradish peroxidase (HRP) antibody was detected using ECL Western Blotting Analysis System (Amersham Biosciences, Piscataway, NJ, USA).

### 4.7. Cell Proliferation Assay

The cell proliferation assay was performed using the Cell Proliferation Reagent WST-1 (Roche, Mannheim, Germany). Cells were seeded into 96-well plates at 2000 cells per well. After TRAIL treatment for indicated time, WST-1 was added with a cell culture medium and incubated for 2 h. Sample absorbance was analyzed using a microplate enzyme-linked immunosorbent assay reader at 490 nm. All experiments were performed at least in triplicate.

### 4.8. Apoptosis Analysis

Annexin V-fluorescein isothiocyanate (FITC)/propidium iodide (PI) staining was performed to identify the induction of apoptosis in PANC-1 cells treated with TRAIL. PANC-1 cells were plated in 60 mm Petri dishes at 2 × 10^5^ cells per dish and cultured overnight. At 12 h after TRAIL treatment, cells were collected and washed with PBS. Cells were stained with Alexa Fluor 488 annexin V and PI using a Vybrant Apoptosis Assay Kit No. 2 (Molecular Probes, Eugene, OR, USA) according to the manufacturer’s protocol. Cells were analyzed using flow cytometry (FACS Calibur, San Jose, CA, USA; Becton Dickinson, Franklin Lakes, NJ, USA) and CellQuest software (Becton Dickinson). Gating was selected using untreated cells to separate most double-negative cells from minor populations of PI or annexin V-FITC-positive cells. Cells that were FITC−/PI− were considered viable, FITC+/PI− cells were considered early apoptotic, and FITC+/PI+ cells were considered nonviable.

### 4.9. Statistical Analysis

An unpaired *t*-test and analysis of variance were used to determine statistical significance. The data are shown as means ± s.d. The differences were considered significant for *p*-values of <0.05. All experiments were replicated at least three times.

## Supplementary Materials

Supplementary materials can be found at http://www.mdpi.com/1422-0067/19/4/1206/s1.

## Abbreviations

PDAC	Pancreatic ductal adenocarcinomas
TRAIL	Tumor necrosis factor-related apoptosis-inducing ligand
FLIP	FADD-like ICE inhibitory proteins
IAP	Inhibitor of apoptosis
miRNAs	MicroRNAs
FasL	Fas Ligand
TNF-α	Tumor Necrosis Factor-Alpha
PARP	Poly ADP ribose polymerase
MECP2	Methyl-CpG Binding Protein 2
EGF	Epidermal Growth Factor
P120RasGAP	P120 RAS GTPase Activating Protein
Rb1	RB Transcriptional Corepressor 1
HBP1	HMG-Box Transcription Factor 1
E2F1	E2F Transcription Factor 1
RBL2	RB Transcriptional Corepressor Like 2
CBFB	Core binding factor beta subunit
PPP3CA	Protein phosphatase 3, catalytic subunit, alpha isozyme
CLINT1	Clathrin interactor 1
HOXB7	Homeobox B7
