# Supplementary material for: MicroRNAs Targeting Caspase-3 and -7 in PANC-1 Cells
Source: Int J Mol Sci. 2018 Apr 16;19(4):1206. doi: 10.3390/ijms19041206 (PMC5979519; doi:10.3390/ijms19041206)
Supplement: Supplementary file 1 [file ijms-19-01206-s001.pdf]

Supplementary Materials

MicroRNAs targeting caspase-3 and -7 in PANC-1 cells

Jong Kook Park [1,*], Andrea I. Doseff [2] and Thomas D. Schmittgen [3,*]

[1] Department of Biomedical Science and Research Institute for Bioscience & Biotechnology, Hallym University, Chuncheon 24252, Republic of Korea

[2] Department of Physiology and Department of Pharmacology and Toxicology, Michigan State University, East Lansing, MI48824, USA; Doseffan@msu.edu

[3] Department of Pharmaceutics, College of Pharmacy, University of Florida, Gainesville, FL 32610, USA

* Correspondence: tschmittgen@cop.ufl.edu (T.D.S.); jkp555@hallym.ac.kr (J.K.P.); Tel.: +1-352-273-8028 (T.D .S.); +82-33-248-2114 (J.K.P.)

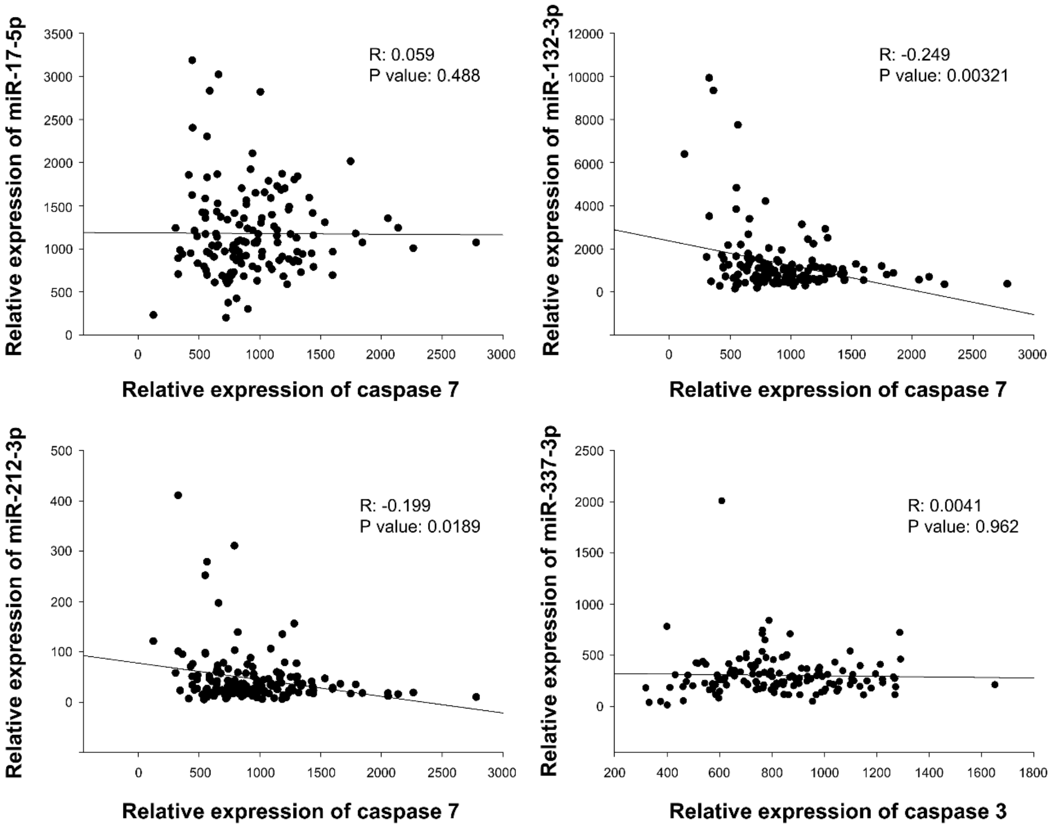

Figure S1. Spearman's correlation between miRNAs and target gene expression. RNA sequencing data of 139 pancreatic ductal adenocarcinoma patients were downloaded from the TCGA data portal. base. The significance of the inverse association between each of these miRNA and mRNA was assessed by the Spearman's correlation coefficient. P values less than 0.05 were considered statistically significant.

Table S1. Clinical data and tumor pathology

Normal pancreas tissues				
Sample No.	Supplier	Age (years)/gender	Cause of death	
N1	Ambion	82/Female	Emphysema	
N2	Ambion	20/Male	Head trauma	
N3	Ambion	71/Male	Cerebral vascular accident	
N4	Ambion	71/Male	Failure to thrive	
Pancreatic adenocarcinoma tissues				
Sample No.	Stage	Differentiation	Age (years)/gender	Adjacent benign
105T	IIb	moderately differentiated	55/Female	O
142T	IV	moderate-poor differentiated	66/Male	O
153T	Ib	moderate-poor differentiated	69/Female	
203T	IIIa	moderate-poor differentiated	64/Male	O
204T	IIb	moderately differentiated	49/Male	
228T	IIa	well differentiated	56/Female	
239T	IV	moderately differentiated	57/Female	
248T	IIb	moderately differentiated	53/Male	
324T	Ib	moderately differentiated	66/Male	O
352T	IIIa	moderate-poor differentiated	82/Male	O
356T	IIb	moderately differentiated	49/Female	O
